# Microscopic Residual Disease After Nasal Squamous Cell Carcinoma Not Visualized on FDG PET-CT: A Case Report

**DOI:** 10.7759/cureus.47451

**Published:** 2023-10-22

**Authors:** Takeshi Tsuda, Takahito Fukusumi, Miyu Saito, Atsuto Kuki, Hidenori Inohara

**Affiliations:** 1 Otorhinolaryngology - Head and Neck Surgery, Osaka University Graduate School of Medicine, Suita-city, Osaka, JPN

**Keywords:** residual cancer, 18f-fluorodeoxyglucose positron emission tomography (18f-fdg pet), squamous cell carcinoma, positron emission tomography, fluorodeoxyglucose, endoscopic sinus surgery, computed tomography

## Abstract

Nasal squamous cell carcinoma (SCC) is rare and aggressive. It often requires combination treatment. Precise post-treatment disease assessment is vital for determining the subsequent management and prognosis. We present the intriguing case of a 52-year-old man with T4bN0M0 stage IVB SCC. Post-treatment fluorodeoxyglucose (FDG) positron emission tomography-computed tomography (PET-CT) findings indicated a complete response; however, microscopic remnants of the cancer were detected during endoscopic sinus surgery. This report underscores the limitations of post-treatment assessment using FDG PET-CT and outpatient endoscopy alone due to these modalities’ potential inability to detect microscopic residual disease. Endoscopic sinus surgery should be incorporated into routine post-treatment assessments of nasal SCC to improve disease detection and guide further treatment. Further large-scale studies are required to confirm these findings.

## Introduction

Nasal squamous cell carcinoma (SCC) is relatively rare. It accounts for less than 1% of all malignancies and approximately 3% of all head and neck cancers [1]. Because nasal SCC is aggressive, combination treatment, usually with surgery, chemotherapy, and radiation therapy, is often provided [2]. However, accurate post-treatment disease assessment, which is essential for determining subsequent treatment and estimating prognosis, remains a challenge in nasal SCC management.

Fluorodeoxyglucose (FDG) positron emission tomography-computed tomography (PET-CT) has emerged as a key technique for post-treatment disease assessment. It is extensively used for staging, response assessment, and surveillance in various cancers and has shown utility in head and neck SCC assessment. FDG PET-CT is highly sensitive and specific for identifying a complete metabolic response to treatment, making it a vital tool in oncology [3]. Nevertheless, its limitations include false positives due to post-treatment changes and difficulties in detecting microscopic residual disease post-treatment, which can lead to underestimation of the disease burden [4].

Herein, we present a case of nasal SCC in which FDG PET-CT indicated a complete metabolic response to chemoradiation therapy, and no residual cancer could be detected via outpatient endoscopy due to the effects of chemoradiation; however, endoscopic sinus surgery revealed microscopic residual cancer. Our report highlights the importance of endoscopic sinus surgery for disease assessment after chemoradiation therapy in patients with nasal SCC, especially when imaging studies and outpatient endoscopy might be insufficient to detect remnant disease.

## Case presentation

**Figure 1:**
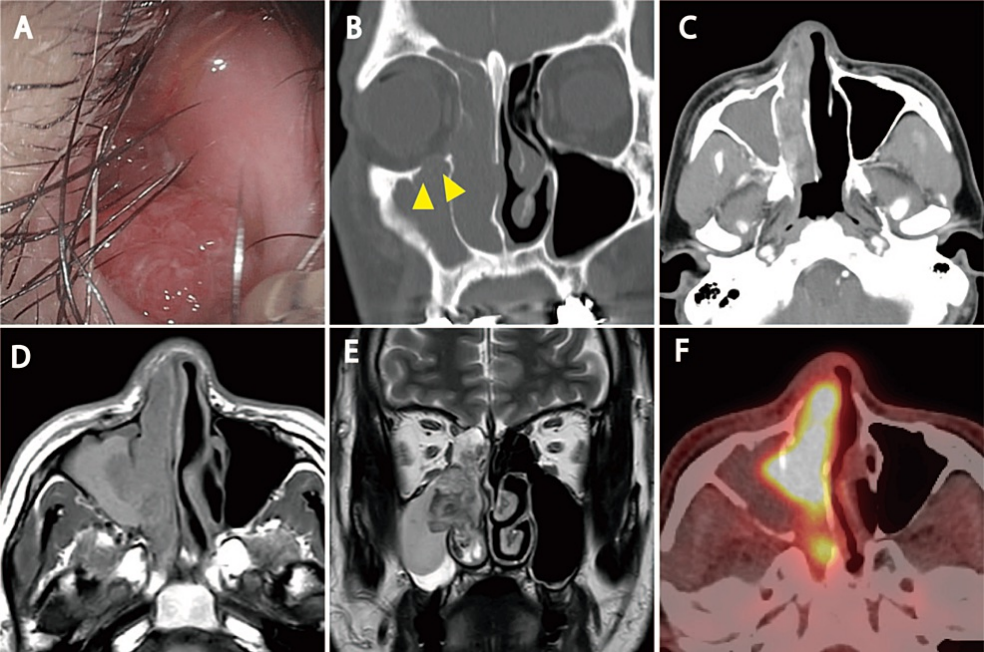
Pre-treatment findings (A) Endonasal fiberoptic endoscopy findings at the initial examination. Hemorrhagic tumors are seen. The tumor fills the right nasal cavity, making it difficult to identify the tumor stem. (B, C) Nasal and paranasal sinus computed tomography (CT) findings. A shadow is seen throughout the right nasal cavity. Contrast effects can be seen in the tumors. Mild osteolysis is seen at the infraorbital wall, suggesting partial orbital invasion (yellow arrowhead). (D), (E) Nasal and paranasal magnetic resonance imaging (MRI) findings. An isointense signal is seen on T1-weighted sequences and an isointense to hyperintense signal is seen on T2-weighted sequences in the tumor area. The tumor has an inhomogeneous internal composition. (F) Fluorodeoxyglucose (FDG) positron emission tomography-CT findings. Strong FDG accumulation is seen in the nasal tumors (maximum standardized uptake value: 17.1), with no apparent accumulation in the rest of the body.

A 52-year-old man with nasal obstruction consulted a general practitioner who identified a tumor in the right nasal cavity and recommended further evaluation at a nearby hospital. At the hospital, a thorough examination and computed tomography (CT) were performed; the results indicated nasal and paranasal malignancies. The patient was referred to our department for further treatment. On arrival at our hospital, we observed a hemorrhagic tumor blocking the right nasal cavity but could not identify its origin (Figure 1A). The patient underwent biopsy and imaging studies, including contrast-enhanced CT (CE-CT), magnetic resonance imaging (MRI), and FDG PET-CT. CE-CT revealed a right nasal cavity mass that extended into the maxillary sinus and exhibited a strong contrast effect. Osteolytic bone was observed at the infraorbital rim adjacent to the tumor and in the orbit (Figures 1B, 1C). MRI revealed heterogeneous enhancement of the mass, with isointense signals on T1-weighted images and isointense to hyperintense signals on T2-weighted images (Figures 1D, 1E). SCC was diagnosed based on the biopsy results. FDG PET-CT revealed FDG accumulation in the tumor (maximum standardized uptake value: 17.1) but no accumulation at other sites; consequently, a clinical diagnosis of T4bN0M0 stage ⅣB nasal SCC was made (Figures 1B-1F).

**Figure 2:**
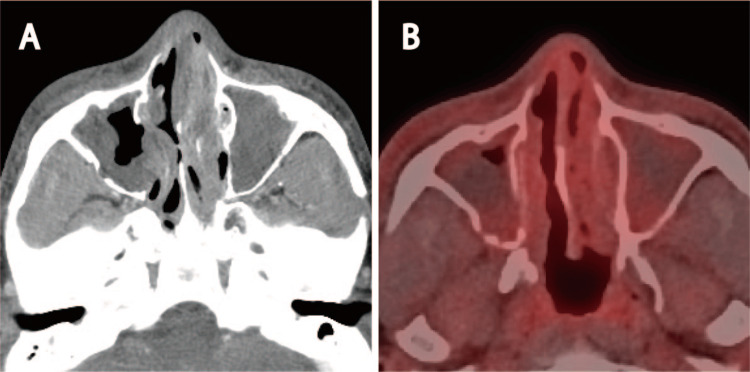
Post-treatment imaging findings (A) Computed tomography (CT) findings after chemoradiation therapy. The primary tumor has shrunk. Shadows are seen at the maxillary and ethmoidal sinuses. (B) Positron emission tomography-CT findings after chemoradiation therapy. The nasal soft tissue shadow has shrunk, and no abnormal hyperaccumulation can be identified. Weak accumulation (maximum standardized uptake value: 2.1) can be observed in the ethmoid and maxillary sinuses, which can be considered to indicate an inflammatory change after treatment.

The patient was admitted to the hospital and concurrently received radiation therapy (total dose of 70 Gy administered at 2 Gy/day across 35 sessions) and chemotherapy (cisplatin 100 mg/m^2^ administered twice). Two months after treatment completion, a residual shadow that was considered to indicate edema was detected in the paranasal sinuses on CE-CT, whereas FDG PET-CT revealed no obvious accumulation. Based on these findings, the patient was considered to have a complete response and complete metabolic response (Figures 2A, 2B).

**Figure 3:**
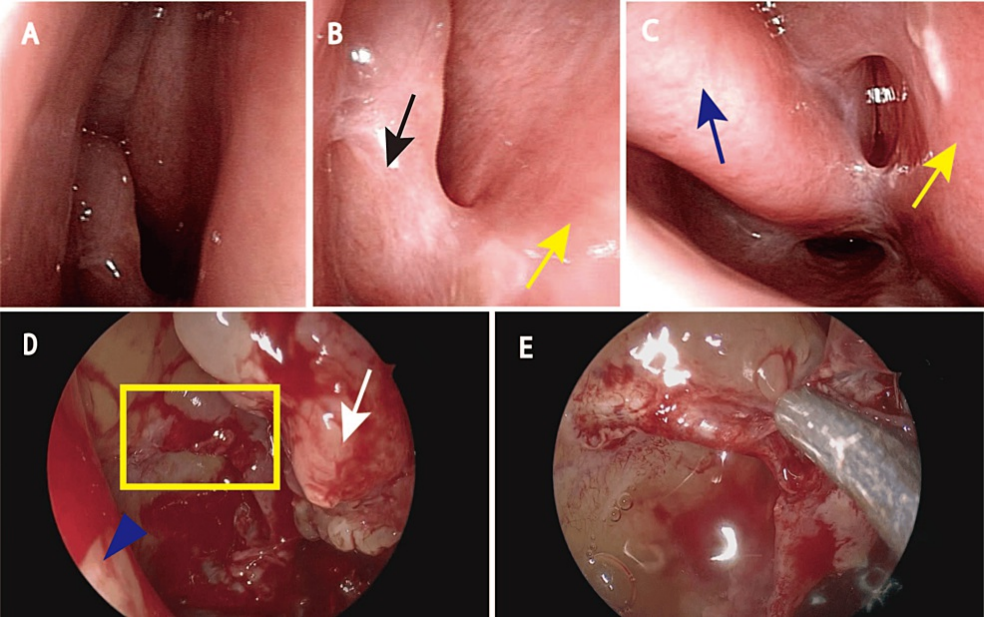
Post-treatment intranasal findings and intraoperative findings (A, B, C) Nasal endoscopy findings at two months post-treatment. Marked edema and adhesions can be seen in the nose. Uncinate process (black arrow), septum (yellow arrow), and inferior turbinate (blue arrow). (D) Intraoperative findings. The nasal area shows edema and adhesions in several locations. Minimal residual carcinoma can be observed on the ethmoid-maxillary plate (yellow box). Inferior turbinate (blue triangle) and middle turbinate (white arrow). (E) Magnified image of a tumor.

An outpatient nasal endoscopy performed to determine treatment efficacy revealed marked edema and multiple adhesions in the nose due to chemoradiation therapy (Figures 3A-3C). As a result, a detailed intranasal evaluation was difficult to perform in an outpatient setting. Therefore, to evaluate for minimal residual malignancy, the patient underwent endoscopic sinus surgery under general anesthesia. Multiple adhesions were noted intraoperatively, with no large tumor remnants noted after dissection. However, the ethmoid-maxillary plate mucosa was slightly irregular, and pathological examination of this area revealed residual SCC (Figures 3D, 3E). Based on these results, the patient decided to undergo adjuvant chemotherapy.

## Discussion

We present a unique case of a 52-year-old man diagnosed with the T4bN0M0 stage IVB SCC of the nasal cavity. Despite imaging findings indicating a complete response to chemoradiation therapy, residual SCC was detected during endoscopic sinus surgery. The novelty of this case primarily lies in the discovery of a microscopic residual malignancy that was missed on both outpatient endoscopy and PET-CT. Our findings emphasize the limitations of currently used diagnostic approaches, particularly in the post-treatment evaluation of nasal SCC [5]. Our observations indicate that incorporating endoscopic sinus surgery into routine post-treatment evaluations may improve the detection of residual disease and guide subsequent management [6].

The role of 18F-FDG PET/CT in pre and post-treatment evaluation in head and neck carcinoma has been emphasized in recent literature [5]. While PET/CT has been instrumental in assessing the metabolic activity of tumors and guiding treatment decisions, its limitations in detecting microscopic residual disease, as seen in our case, are evident. This is consistent with the findings of Tantiwongkosi et al., who highlighted the significance of PET/CT in the management of head and neck cancer but also acknowledged its limitations in certain scenarios [7].

Furthermore, our case underscores the importance of a comprehensive approach to post-treatment evaluation. While imaging techniques like PET-CT offer valuable insights, they cannot replace the detailed assessment provided by endoscopic evaluations [6]. As noted in our case, patients with nasal cavity cancer often have significant edema and adhesions after chemoradiotherapy, making the evaluation of minimal residual lesions via outpatient endoscopy challenging. This observation aligns with the findings from the study on the role of 18F-FDG PET/CT in the evaluation of head and neck carcinoma, which emphasized the importance of combining imaging with clinical evaluations for a more accurate assessment.

Moreover, the challenges in detecting residual disease post-treatment are not unique to nasal SCC. The complexities in assessing head and neck malignancies, especially in the post-treatment phase, have been highlighted in various studies [5,8]. These studies emphasize the need for a multi-modal approach, combining imaging, clinical evaluations, and surgical interventions, to ensure comprehensive patient care.

One limitation of our study is that our findings are based on a single case. Although this case provides valuable insights into the potential gaps in nasal SCC assessment, further studies with larger sample sizes are needed to validate our observations. Given the increasing prevalence of head and neck cancers and the evolving landscape of diagnostic and therapeutic modalities, it is imperative to continuously re-evaluate and refine our approaches to ensure optimal patient outcomes.

## Conclusions

Extensive fibrotic adhere and edematous alterations are observed in the nasal cavity subsequent to combined chemoradiotherapy for nasal SCC. PET-CT, conventionally instrumental in detecting neoplastic processes, presents challenges, encompassing potential false-positive results stemming from therapy-induced inflammation, and the intricacy in assessing diminutive lesions. Relying solely on imaging studies for the post-treatment assessment of nasal SCC has disadvantages. Pathological examination is crucial for assessing residual lesions after treatment and incorporating endoscopic sinus surgery (ESS) into cases in which outpatient endoscopy does not provide adequate local evaluation can be beneficial, particularly for detecting microscopic residual disease not identified by other modalities. Based on the above, it was considered important to combine imaging tests, such as PET-CT and MRI, with pathological tests for the evaluation of residual lesions after treatment and to add evaluation by ESS as necessary.
